# Qualitative methodology: considerations and singularities regarding the implementation of person-centered interventions

**DOI:** 10.1590/0034-7167.2024770301

**Published:** 2024-05-13

**Authors:** Ellen Synthia Fernandes de Oliveira, Maria Helena Presado, Cristina Lavareda Baixinho

**Affiliations:** I Universidade Federal de Goiás, Docente do Programa de Pós-Graduação em Saúde Coletiva. Goiânia, Goiás, Brazil; II Escola Superior de Enfermagem de Lisboa, Docente de Enfermagem. Lisboa, Portugal; III Centro de Investigação, Inovação e Desenvolvimento em Enfermagem de Lisboa. Lisboa, Portugal; IV Centro de Inovação em Tecnologias e Cuidados de Saúde. Leiria, Portugal

Qualitative health research allows for an in-depth understanding of how a person experiences different health transitions, their relationship with healthcare professionals and their passage through different clinical practice environments. At the same time, it has the potential to support professionals’ clinical decision-making and to empower citizens in their autonomy and responsibility for the health-disease process, inserted in their life project^(1)^.

Another aspect of qualitative methods is their usefulness for designing interventions and actively involving, from the beginning of the study, people in the co-construction of research so that it translates into results sensitive to individual needs. Furthermore, it is important to understand issues related to implementation, the theorization of action mechanisms, the understanding of how the context influences and the acceptability of interventions^(2)^.

Interventions in health in general and in nursing, in particular, are complex and are always in permanent interaction with a multiplicity of factors that are difficult to measure and replicate, because care needs, difficulties in adhering to therapy(ies), expectations and the human experience itself are individual, always expressed in the first person. Some authors argue that the interpretivist methodology is appropriate for designing, developing and implementing complex interventions, because health service interventions are represented as consistent, objective and static, but in fact involve interpretation in the way they are designed, validated, implemented and adopted by people^(2)^.

In this line of thought, if we reflect that the person-centered care approach implies the “adaptation” of evidence to each person’s needs and preferences, it is clear that the methods used in the design of interventions must allow to hear their voice, know the coping strategies for managing symptoms and decisions regarding a set of complex issues^(3,4)^, which reinforces the importance of this paradigm and its use in different phases of the study to ensure that the intervention is acceptable, viable, meaningful and engaging for the person(s)^(2,3,4)^.

The SARS-CoV-2 pandemic and the need to (re)organize healthcare systems to access a greater number of patients and meet the necessary demands for hospitalization were challenges to the growing demand for new interventions, especially because healthcare professionals are increasingly dealing with complex problems, such as integration of health and socioeconomic support needs, new risks associated with lifestyle and use of e-health technology^(4)^. These changes are a challenge for focused care and the implementation of safe, quality and even cost-effective interventions, since a precarious intervention design can waste public resources through expensive, unreasonable and non-problem/need-oriented assessments, leading to ineffective action^(4)^.

Therefore, healthcare interventions must be carefully designed and developed in order to enhance the response to health problems so that they are adjusted and effective in their purpose in order to be implemented as planned, promoting the development of healthcare and societies by guaranteeing the best care, based on best evidence, with respect for preferences/needs appropriate to the context and culture^(1,2,3,4)^.

Considering the above, we reinforced that qualitative research and more collaborative/participatory research methods, developed through a systematic and structured process, allow from designing to implementing more effective interventions in responding to health problems, as they are based on the understanding of the problem and designed to respond to how this same problem is experienced by the target population in the context of interest^(3,4)^.

## References

[1] Oliveira ESF, Baixinho CL, Presado MHCV. (2019). Qualitative research in health: a reflexive approach. Rev Bras Enferm.

[2] Thirsk LM, Clark AM. (2017). Using qualitative research for complex interventions: the contributions of hermeneutics. Int J Qual Methods.

[3] Yardley L, Bradbury K, Morrison L, Camic PM. (2021). Qualitative research in psychology: expanding perspectives in methodology and design.

[4] O’Cathain A, Croot L, Duncan E. (2019). Guidance on how to develop complex interventions to improve health and healthcare. BMJ Open.

